# Asymmetry of Auditory-Motor Speech Processing is Determined by Language Experience

**DOI:** 10.1523/JNEUROSCI.1977-20.2020

**Published:** 2021-02-03

**Authors:** Ding-lan Tang, Riikka Möttönen, Salomi S. Asaridou, Kate E. Watkins

**Affiliations:** ^1^Wellcome Centre for Integrative Neuroimaging, Department of Experimental Psychology, University of Oxford, Oxford, OX2 6GG, United Kingdom; ^2^School of Psychology, University of Nottingham, Nottingham, NG7 2RD, United Kingdom

**Keywords:** auditory-motor interaction, hemispheric lateralization, mismatch negativity, tonal language processing, transcranial magnetic stimulation

## Abstract

Speech processing relies on interactions between auditory and motor systems and is asymmetrically organized in the human brain. The left auditory system is specialized for processing of phonemes, whereas the right is specialized for processing of pitch changes in speech affecting prosody. In speakers of tonal languages, however, processing of pitch (i.e., tone) changes that alter word meaning is left-lateralized indicating that linguistic function and language experience shape speech processing asymmetries. Here, we investigated the asymmetry of motor contributions to auditory speech processing in male and female speakers of tonal and non-tonal languages. We temporarily disrupted the right or left speech motor cortex using transcranial magnetic stimulation (TMS) and measured the impact of these disruptions on auditory discrimination (mismatch negativity; MMN) responses to phoneme and tone changes in sequences of syllables using electroencephalography (EEG). We found that the effect of motor disruptions on processing of tone changes differed between language groups: disruption of the right speech motor cortex suppressed responses to tone changes in non-tonal language speakers, whereas disruption of the left speech motor cortex suppressed responses to tone changes in tonal language speakers. In non-tonal language speakers, the effects of disruption of left speech motor cortex on responses to tone changes were inconclusive. For phoneme changes, disruption of left but not right speech motor cortex suppressed responses in both language groups. We conclude that the contributions of the right and left speech motor cortex to auditory speech processing are determined by the functional roles of acoustic cues in the listener's native language.

**SIGNIFICANCE STATEMENT** The principles underlying hemispheric asymmetries of auditory speech processing remain debated. The asymmetry of processing of speech sounds is affected by low-level acoustic cues, but also by their linguistic function. By combining transcranial magnetic stimulation (TMS) and electroencephalography (EEG), we investigated the asymmetry of motor contributions to auditory speech processing in tonal and non-tonal language speakers. We provide causal evidence that the functional role of the acoustic cues in the listener's native language affects the asymmetry of motor influences on auditory speech discrimination ability [indexed by mismatch negativity (MMN) responses]. Lateralized top-down motor influences can affect asymmetry of speech processing in the auditory system.

## Introduction

Over the past two decades, a growing number of studies have emphasized the importance of interactions between auditory and motor systems for speech perception (Skipper et al., 2017; Liebenthal and Möttönen, 2018). The dorsal pathway connecting auditory and motor systems is strongly left-lateralized for phonemic processing (Hickok and Poeppel, 2007). In contrast, auditory-motor processing of prosody is lateralized to the right hemisphere (Ethofer et al., 2006; Wildgruber et al., 2006; Sammler et al., 2015).

Asymmetry in auditory processing is thought to reflect differences in the temporal and spectral sensitivities of the left and right auditory cortex to acoustic inputs (e.g. Zatorre and Belin, 2001). An extensive body of work indicates that the left auditory cortex is specialized for processing rapidly changing temporal information, which is necessary to resolve phonemic changes, i.e., to discriminate small acoustic differences in speech sounds that alter word meaning. In contrast, the right auditory cortex is sensitive to more slowly changing auditory information and the spectral content of the same acoustic signals, which is necessary to discriminate prosodic changes in speech, such as the use of pitch for stress, or to convey emotion (Meyer et al., 2002). Although this view has gained lots of support, it should be noted that it has been primarily tested in speakers of non-tonal languages (Boemio et al., 2005; Jamison et al., 2006; Flinker et al., 2019; Albouy et al., 2020).

For as many as 70% of the world's languages, suprasegmental speech elements such as changes in pitch (i.e., tones) are also used to alter word meaning (Yip, 2002). For example, in Mandarin Chinese, the word “ma” produced with a high-level tone (ma1) means “mother” and when produced with a falling-rising tone (ma3) means “horse.” Auditory processing of these lexical tones in tonal language speakers is also left-lateralized (Gandour et al., 2000; Hsieh et al., 2001; Klein et al., 2001), even though it does not require rapid temporal processing of the acoustic signal. In this case, the left lateralization reflects the linguistic function of the acoustic signals and thereby demonstrates how language experience can alter hemispheric lateralization of speech processing.

It is likely therefore that asymmetry in speech processing is determined by bottom-up properties in the acoustic signal as well as by top-down influences (Zatorre and Gandour, 2008; Albouy et al., 2020). We hypothesized that one such influence could come from the speech motor system. Disruption of the left speech motor cortex by transcranial magnetic stimulation (TMS) affects performance on speech discrimination tasks (Möttönen and Watkins, 2009; Möttönen et al., 2014b; Smalle et al., 2015) and electroencephalographic (EEG) measures of automatic phoneme discrimination, namely the mismatch negativity (MMN; Möttönen et al., 2013). Less is known about the contribution of the right speech motor cortex to auditory speech processing and the between-hemisphere asymmetry of such motor contributions.

Here, we aimed to determine whether speech processing in the auditory system is affected by lateralized interactions with the motor system. We investigated the asymmetry of auditory-motor speech processing in both tonal and non-tonal language groups capitalizing on the fact that the same low-level acoustic signal (pitch) serves different linguistic functions in each.

We used low-frequency repetitive TMS (rTMS) to temporarily disrupt the speech motor cortex and EEG to record MMN responses to changes in a sequence of frequent “da” syllables (da1) that were either a phoneme (ba1) or a tone change (da4). If hemispheric differences in motor contributions to auditory processing relate to low-level acoustics, then disruption of the right speech motor cortex should modulate tone processing and disruption of the left speech motor cortex should modulate phoneme processing in both language groups equally. In contrast, if these differences arise because of the functional roles of the acoustic cues, which differ between tonal and non-tonal languages for tone processing, then the effect of motor disruptions on processing of tone changes would differ between tonal and non-tonal language groups.

## Materials and Methods

### 

#### Participants

We recruited 32 adult human participants (16 male, 16 female) who were speakers of non-tonal languages and aged between 18 and 35 years and 32 tonal language speakers (16 male, 16 female) aged between 18 and 34 years. None of the 64 participants had received any formal musical training that had lasted longer than two years. The native languages of the group of non-tonal language speakers were: English (21), French (2), Spanish (2), Czech (2), German (1), Italian (1), Romanian (1), Polish (1), and Dutch (1). None of the non-tonal language speakers had ever learned a tonal language but most had learned another language to varying levels of proficiency. All the tonal language speakers were native speakers of Mandarin and had at least a basic level of English; 29 were studying at an English-speaking university, and all were living in the United Kingdom. The ages at which they first started to learn English ranged from 7 to 15 years old. Male and female participants in each language group were randomly assigned to receive either left or right hemisphere stimulation, so that groups were gender balanced. All participants were right-handed and reported no hearing problems and no personal or family history of seizures or other neurologic disorders. The Central University (of Oxford) Research Ethics Committee approved the experimental protocol and participants gave informed consent. EEG data from two non-tonal language speakers (both native English speakers; one male, one female) were excluded from the analyses because their mean MMN amplitudes were extreme (>3 SDs from the group mean MMN amplitude). The mean ages and gender balance for the four groups that contributed data to the analysis are as follows: non-tonal language, left-hemisphere stimulation group: mean age 21.60 years, eight males, seven females; non-tonal language, right hemisphere stimulation group: mean age 22.60 years, seven males, eight females; tonal language, left-hemisphere stimulation group: mean age 25.69 years, eight males, eight females; tonal language, right hemisphere stimulation group: mean age 26.13 years, eight males, eight females. There was no significant difference among groups in terms of age.

#### Procedure

For all participants, EEG during a 16.5-min oddball sequence was recorded during two sessions: a no-TMS baseline and a post-TMS session. The oddball sound sequence comprised infrequent tone and phoneme changes, which were expected to elicit MMN responses. During no-TMS baseline sessions, event-related potentials (ERPs) were also recorded to two control sequences (see Stimuli below). The order of presentation of oddball and control sequences was counterbalanced. Participants watched a silent movie and were instructed to ignore the sound sequences during all recordings. The order of no-TMS and post-TMS sessions was counterbalanced: half participants started with the no-TMS session followed by rTMS and then an immediate post-TMS session, while the other half received rTMS first, the post-TMS session immediately after, and then a no-TMS session at least 50 min later. TMS involved 15 min of low-frequency (0.6 Hz) subthreshold repetitive stimulation over the lip representation (henceforth, speech motor cortex) in either the left or the right primary motor cortex. This protocol temporarily inhibits excitability in the target representation for a further 15 min approximately (Möttönen and Watkins, 2009).

#### Stimuli

**Figure 1.**
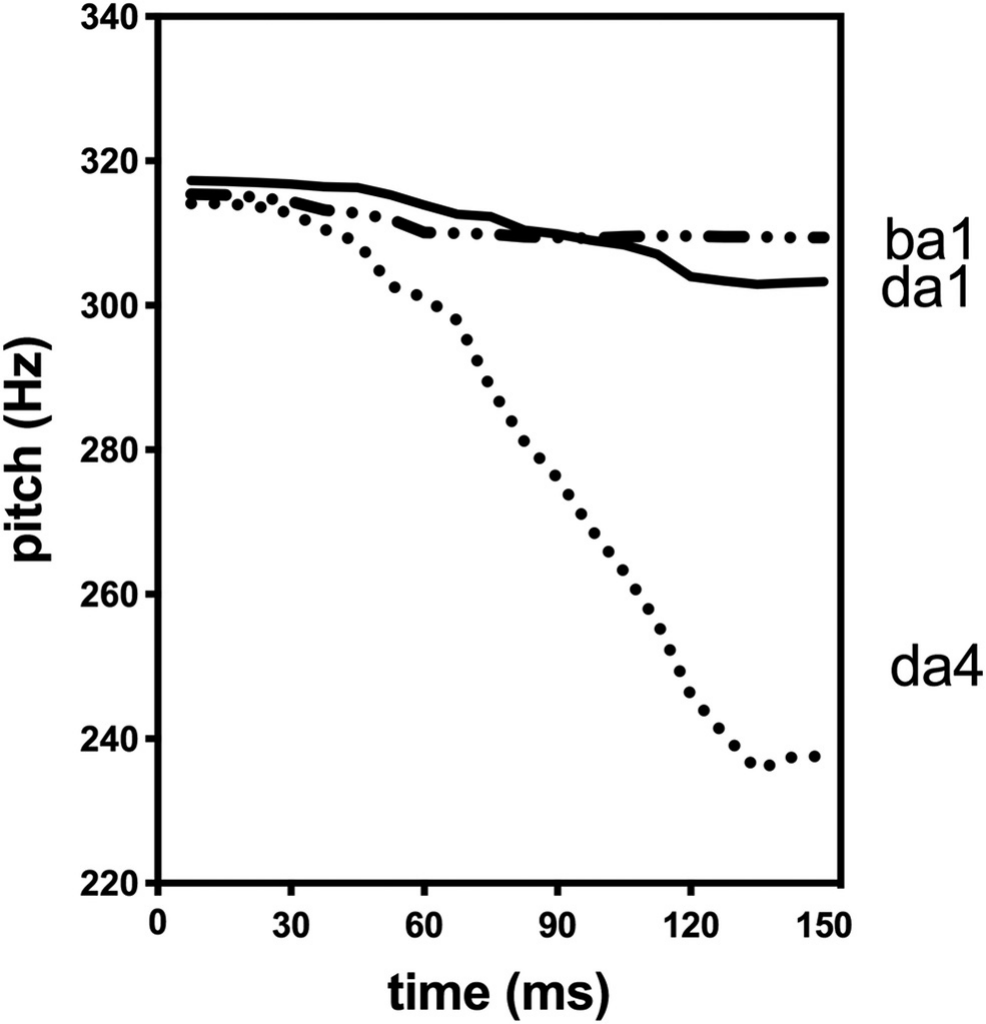
The trajectories of the fundamental frequency (F0) of the three syllables used in the current study. Ba1 is a “ba” syllable with tone 1, which is a high-level tone; da1 is a “da” syllable also with tone 1; da4 is the same “da” syllable with tone 4, which is a falling tone.

The oddball sequence contained a total of 1800 stimuli, including infrequent 'ba1' and 'da4' (probability = 0.1 for each) and frequent 'da1' syllables (probability = 0.8). Control sequences consisted of 400 repetitions each of the infrequent syllables in the oddball sequence: two control sequences, one for 'ba1' and one for 'da4'. Syllables 'da1' (tone 1: high-level tone) and 'da4' (tone 4: high falling tone) shared the same phoneme but carried different tone contours, while 'da1' and 'ba1' had the same high-level tone (tone 1) but contained different phoneme information. The original stimuli ('da1,' 'da4,' 'ba1') were recorded from a female native Mandarin speaker. Using Praat software (Institute of Phonetic Sciences, University of Amsterdam, The Netherlands), we digitally normalized them to equal durations (150 ms) and matched them for fundamental frequency (F0) for the first 35 ms of the syllable. The 'da4' syllable differed from the other two in the F0 trajectory after 35 ms, which is clearly seen in Figure 1. The interstimulus interval was 400 ms in all sequences. Stimulus presentation was controlled via custom scripts in MATLAB (MathWorks). Participants wore TMS-compatible insert earphones; stimuli were played at a comfortable listening level ∼70 dB.

#### TMS

All TMS pulses were monophasic, generated by two Magstim 200s and delivered through a 70-mm figure-8 coil connected through a BiStim module.

Before rTMS, single TMS pulses were used to localize the lip representation, following the procedure previously described (Möttönen et al., 2014a). The coil was placed tangential to the skull to induce a current flow from posterior to anterior under the junction of the two wings of the figure-8 coil. The position and orientation of the coil over the lateral scalp (starting with the handle held horizontally) was adjusted until a robust motor-evoked potential (MEP) was observed in the contralateral target muscle. Then, once the target area had been located, we established the active motor threshold, that is, the minimum intensity at which TMS elicited at least five out of 10 MEPs with an amplitude of at least 200 μV when the target muscle was contracted at 20–30% of the maximum. The mean active motor threshold (percentage of maximum stimulator output, ±SD) for the left and right stimulation groups, respectively, was 63.1% (±7.8%) and 62.1% (±7.1%) in the tonal language speakers, and 58.8% (±6.1%) and 57.47% (±7.4%) in the non-tonal speakers (no significant differences among the four groups; *F*_(3,58)_ = 2.71, *p* = 0.101). The intensity of each participant's active motor threshold was used for 15-min rTMS while the muscles were relaxed (hence the stimulation was subthreshold). In total, each participant received 600 pulses at 0.6 Hz (rTMS), and between 30 and 60 single pulses for thresholding and localizing. During rTMS, participants watched a silent nature documentary and were instructed to stay still. Electromyography (EMG) recordings were carefully monitored throughout stimulation to ensure that muscles were relaxed, and no MEPs were evoked in the lip muscles. The coil was changed halfway through rTMS to prevent overheating.

#### EMG recordings

Disposable electrodes were attached on the right or left orbicularis oris muscle (the upper and lower lip contralateral to the stimulation site) and forehead (ground) to record EMG signals. The EMG signals were amplified, sampled at 5000 Hz, filtered (1-Hz to 1-kHz bandpass) using a CED 1902 amplifier, a CED 1401 analog-to-digital converter, and a PC running Spike2 (Cambridge Electronic Design). A power bar displayed on a computer screen allowed participants to practice producing a constant level of contraction of the lips (20–30% of the maximum).

#### EEG recording parameters

We acquired EEG using Synamps amplifiers and Curry 7 data acquisition software. We used a custom electrode cap with 11 electrodes (Fz, FCz, Cz, CPz, Pz, F1, F2, P1, P2, left and right mastoids), which allowed us to position the coil over sites of interest on the lateral convexity of the scalp. The ground and reference electrodes were placed on the right upper arm and the tip of the nose, respectively. During acquisition, data were low-pass filtered (400-Hz cutoff), digitized at 1000 Hz, and stored for offline analysis. Electrode impedances were reduced below 10 kΩ before recording.

#### EEG data analyses

EEG signals were preprocessed using the EEGLab Toolbox (Delorme and Makeig, 2004). The raw data (down sampled to 500 Hz) were first re-referenced to the mean of the two mastoids to improve the signal-to-noise ratio of the MMN responses. Then the data were digitally filtered (low-pass filter of 30 Hz, high-pass filter of 1 Hz), baseline corrected, and segmented into epochs of 400 ms comprising a 100-ms prestimulus baseline and a 300-ms interval after the onset of the stimuli. The epochs containing amplitude fluctuations exceeding ± 70 μV were considered as artifacts and removed before averaging. In addition, the epochs for the first 10 stimuli in each sequence and for the first standard stimulus after each deviant were removed.

Both tone and phoneme changes were expected to elicit MMNs, a fronto-central negative component with sources primarily in the left and right auditory cortex, which reflects the automatic detection of a deviant stimulus in a passive auditory odd-ball paradigm (Näätänen et al., 1997, 2001; Winkler et al., 1999). The amplitude of the MMN provides an index of an individual's ability to detect the change in the stimulus sequence, which requires discrimination between deviant and standard stimuli (Näätänen, 2008). For example, phonemic changes of the native language that are easily detectable elicit large MMNs, whereas phonemic changes that do not exist in the native language are hard to detect and elicit smaller MMNs (Näätänen et al., 1997).

For the baseline (no TMS) sessions, MMN responses were calculated in two ways: the traditional method (subtracting the ERPs evoked by the frequent stimuli from those evoked by the infrequent stimuli in the oddball sequence), and the same-stimulus method (subtracting ERPs evoked by the stimuli presented frequently in the control sequence from those evoked by identical sounds presented infrequently in the oddball sequences). The advantage of the traditional method is that the responses are recorded for frequent and infrequent stimuli during the same sequence. The disadvantage is that we are comparing responses to sounds that differ acoustically. The same-stimulus method gets around this potential confound by comparing the response to acoustically identical stimuli presented either in the control sequence (i.e., with other identical stimuli) or in the oddball sequence as an infrequent sound (i.e., with other acoustically different stimuli). Identity MMN responses obtained using the same-stimulus method therefore reflect differences because of the automatic discrimination of speech sounds presented infrequently in the context of more frequent but acoustically different speech sounds (Jacobsen and Schröger, 2001, 2003; Kujala et al., 2007). Identity MMN responses were calculated for the baseline sessions to confirm that the responses were because of discrimination of speech sounds (i.e., that they were genuine). For the post-TMS sessions, MMN responses were calculated using the traditional method only as there was insufficient time to obtain control sequences during the TMS-induced disruption, which is thought to last <20 min.

#### Statistical analyses

Statistical analyses were preplanned and followed the methods used in Möttönen et al. (2013), thereby allowing comparison between studies. MMN responses calculated using both the traditional or the same-stimulus (identity MMNs) method were maximal at the FCz electrode, as seen previously (Näätänen et al., 1997, 2001; Möttönen et al., 2013). MMN responses at FCz were selected for statistical analyses therefore; data for all electrodes are available at https://osf.io/en7uq/.

To evaluate whether the TMS-induced disruption of the motor cortex modulated MMN responses, we compared MMN responses (calculated using the traditional method) in the no-TMS and post-TMS conditions. Paired *t* tests (two-tailed) were calculated at each time point from 0 to 300 ms after the onset of the syllable to determine whether any portion of the MMN responses differed significantly between conditions and the latencies of these differences. To control for false positives, the MMN differences were considered significant when p-values were lower than 0.05 for 10 (=20 ms) or more consecutive time points (Guthrie and Buchwald, 1991; Möttönen et al., 2013). These analyses were conducted for the phoneme and tone contrasts and each language group separately.

In a separate set of analyses, mean MMN amplitudes were calculated as the mean voltage across a 40-ms window centered at the peak latency for each participant. Peak amplitudes were calculated in the same way for the identity MMNs obtained in the baseline (no-TMS) session. To test whether significant MMN responses were elicited in the no-TMS and post-TMS sessions, we compared the peak amplitudes against zero (no change) using one-sample *t* tests. The amplitudes and latencies of these responses were compared between groups using two-sample *t* tests. To test the influence of language experience on the TMS-induced disruptions of speech motor cortex on the peak amplitude of the MMN responses elicited by phoneme and tone contrasts, separate repeated measures ANOVAs were conducted with TMS (no-TMS vs post-TMS) as a within-subjects factor and language group (tonal vs non-tonal language speakers) as a between-subjects factor. Interactions were explored using *post hoc* paired *t* tests (no-TMS vs post-TMS) for each group separately and to provide Cohen's *d* effect sizes for future studies.

## Results

### Effects of TMS-induced disruption in the right speech motor cortex

We stimulated the right speech motor cortex in 15 non-tonal language speakers and 16 tonal language (Mandarin) speakers and tested the effect of this disruption on discrimination of tone and phoneme contrasts measured by the MMN. We recorded EEG while participants listened to an oddball sequence in no-TMS (baseline) and post-TMS sessions. ERPs to frequent sounds ('da4') were subtracted from responses to infrequent phoneme ('ba1') and tone ('da4') changes to obtain the MMN responses. To evaluate the effect of TMS-induced disruption on these measures of phoneme and tone discrimination in each language group, we first compared the MMN responses in the two sessions using sequential paired *t* tests. This analysis determined the latencies of any significant differences.

**Figure 2.**
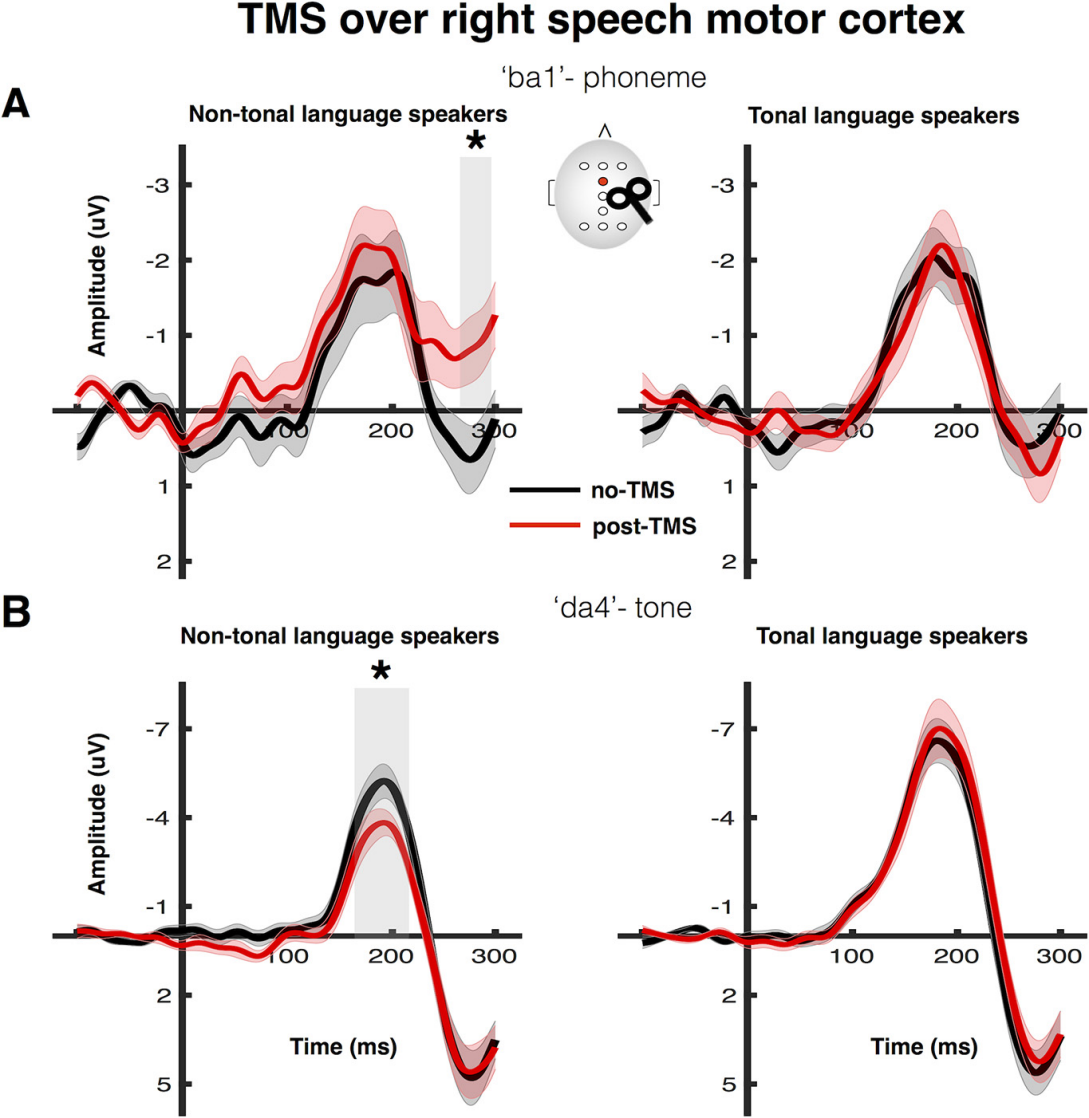
Effect of rTMS over right speech motor cortex on MMN responses elicited by the phoneme (***A***) and tone (***B***) changes in tonal and non-tonal language speakers. The MMN responses recorded from the FCz electrode were obtained by subtracting the responses to frequent 'da1' syllable from the responses to infrequent 'ba1' (***A***) or 'da4' (***B***) syllables. Shaded regions around the MMN responses indicate SEM (solid line) across participants. The gray rectangle indicates the time windows during which the no-TMS (black) and post-TMS (red) ERPs differed significantly from each other (sequential *t* tests); *p < 0.05 (two-tailed). Note the different scales used for ***A***, ***B***; MMN responses to the tone change were robustly higher than for the phoneme change.

TMS-induced disruption of the right speech motor cortex had no effect on the MMN response elicited by the phoneme change (Fig. 2*A*, 'ba1') in either the tonal or the non-tonal language group. In contrast, MMN responses elicited by the tone change were significantly suppressed by TMS-induced disruption of the right speech motor cortex at a latency of 158–230 ms after stimulus onset in the non-tonal language group but had no effect on these responses in the tonal language group at any latency (Fig. 2*B*, 'da4'). In the non-tonal language group there was a significant difference between no-TMS and post-TMS responses to the phoneme change at a latency of 264–300 ms (Fig. 2*A*, 'ba1'); this late discriminatory negativity occurred after the MMN response and possibly indicates prolonged processing because of the right hemisphere disruption (Cheour et al., 2001).

Peak amplitudes for MMN responses were calculated by averaging the voltage across a 40-ms window centered at the peak latency in each participant for each contrast. We used one-sample *t* tests against zero to confirm that significant MMN responses were elicited in both no-TMS and post-TMS sessions by the phoneme and tone changes in both groups (Table 1). Comparison of MMN responses calculated using the traditional method with identity MMNs calculated using the same-stimulus method, confirmed that these changes were because of discrimination of speech sounds (Tables 1, 2). Furthermore, during the no-TMS session, we confirmed that the latencies and amplitudes of the MMN responses elicited by either tone or phoneme changes did not differ between language groups (two-sample *t* tests: all *p* > 0.05).

**Table 1.** MMN Peak latencies (ms) and mean amplitudes (SEM, μV) at FCz

	Latency	No-TMS Amplitude	*t*	Latency	Post-TMS Amplitude	*t*
TMS over right speech motor cortex						
Non-tonal language group, *n* = 15						
'ba1'	182.9	−2.25 (0.49)	4.58	194.3	−2.68 (0.51)	5.24
'da4'	191.7	−5.40 (0.38)	14.39	191.1	−4.00 (0.41)	9.69
Tonal language group, n = 16						
'ba1'	174.0	−2.29 (0.35)	6.52	175.8	−2.43 (0.43)	5.70
'da4'	182.5	−6.91 (0.63)	10.93	183.3	−7.16 (0.84)	8.57
TMS over left speech motor cortex						
Non-tonal language group, *n* = 15						
'ba1'	182.8	−2.97 (0.32)	9.35	190.7	−2.19 (0.27)	8.07
'da4'	189.7	−5.80 (0.59)	9.80	192.0	−4.86 (0.70)	6.92
Tonal language group, *n* = 16						
'ba1'	181.5	−2.63 (0.30)	8.69	199.4-	−1.75 (0.32)	5.39
'da4'	191.6	−6.61 (0.70)	9.47	187.6	−4.91 (0.66)	7.41

The MMN amplitudes were calculated by averaging the responses across a 40-ms window centered at this peak latency and were compared with zero using two-tailed *t* tests. All *t* test results are significant with *p* < 0.001.

**Table 2.** Identity MMN peak latencies (ms) and mean amplitudes (μV, ±SEM) at FCz

	Latency	Amplitude	*t*	*p*
TMS over right speech motor cortex				
Non-tonal language speakers, n = 15				
'ba1'	191.9	−1.37 (0.49)	2.81	<0.05
'da4'	192.3	−6.01 (0.42)	14.25	<0.001
Tonal language speakers, n = 16				
'ba1'	201.4	−2.29 (0.49)	4.69	<0.001
'da4'	183.4	−7.50 (0.69)	10.82	<0.001
TMS over left speech motor cortex				
Non-tonal language speakers, *n* = 15				
'ba1'	210.4	−2.20 (0.30)	7.41	<0.001
'da4'	188.8	−7.24 (0.71)	10.21	<0.001
Tonal language speakers, *n* = 16				
'ba1'	204.6	−2.64 (0.41)	6.34	<0.001
'da4'	193.3	−7.62 (0.68)	11.28	<0.001

The identity MMN responses were calculated by subtracting ERPs evoked by the stimuli presented frequently (probability = 1.0) in the control sequence from those evoked by identical sounds presented infrequently (probability = 0.1) in the oddball sequence (with no TMS). Mean amplitudes were calculated by averaging the responses across a 40-ms window centered at this peak latency and were compared with zero using two-tailed *t* tests.

**Figure 3.**
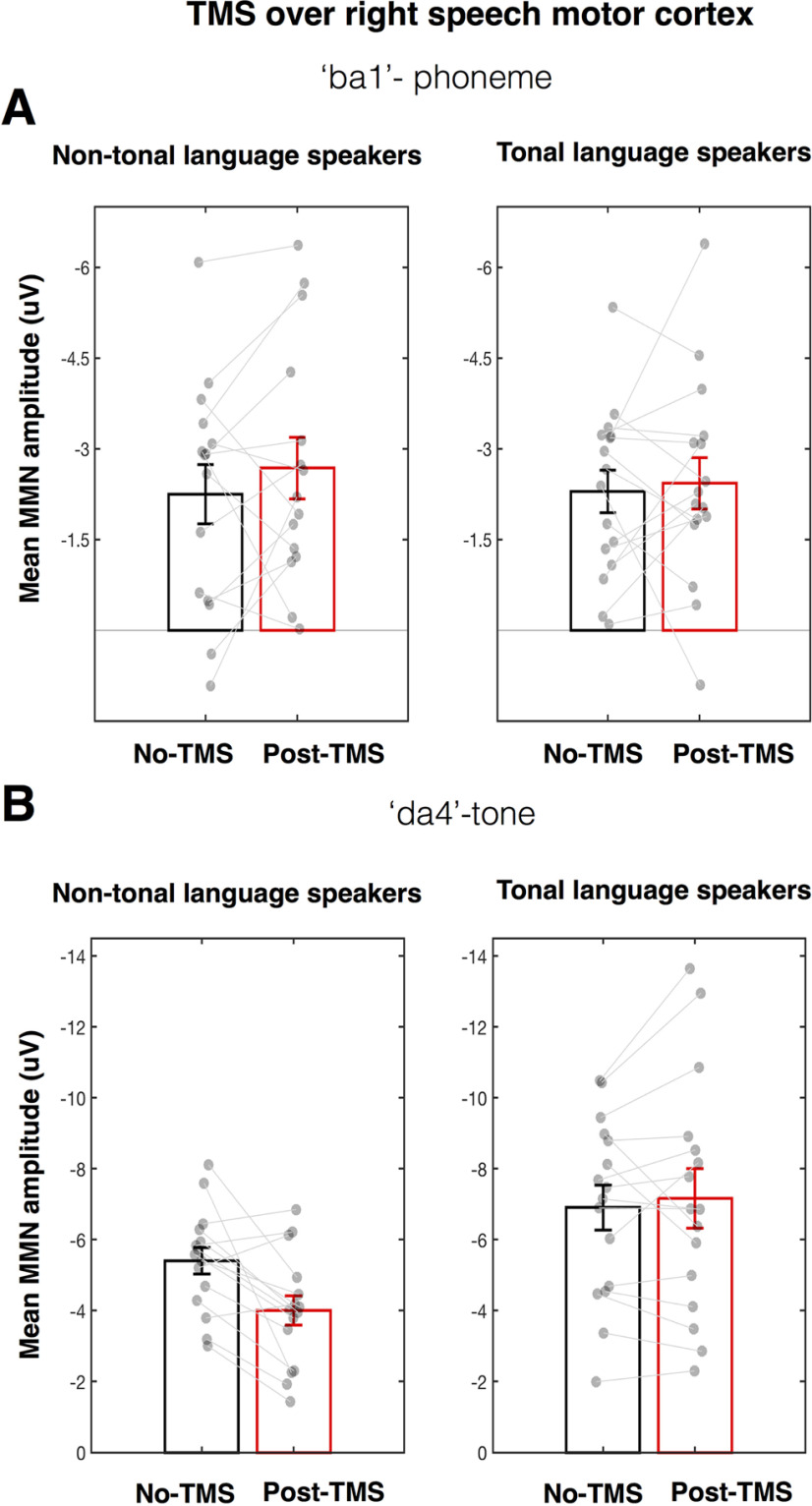
Effect of rTMS over the right speech motor cortex on amplitudes of MMN responses elicited by the phoneme (***A***) and tone (***B***) changes in tonal and non-tonal language speakers. The gray circles show the mean amplitudes of MMN responses obtained during no-TMS and post-TMS recordings for individual participants connected by a gray line. The group mean MMN amplitudes during no-TMS and post-TMS recordings are shown in black and red unfilled bars, respectively. Error bars represent SEM.

To test whether the effects of TMS-induced disruption of the right speech motor cortex on the MMN responses were sensitive to the language experience of the participants, we compared the groups using two-way ANOVAs for the phoneme and tone contrasts separately. For the MMN responses elicited by the phoneme change, the TMS-induced disruption in the right speech motor cortex had no effect in either language group (i.e., the main effect of TMS was not significant: *F*_(1,29)_ = 1.02, *p* = 0.322, η_p_^2^ = 0.03; see Fig. 3*A*; the main effect of group was also not significant: *F*_(1,29)_ = 0.03, *p* = 0.858, η_p_^2^ < 0.01). For the MMN responses elicited by the tone change, there was a significant main effect of group (*F*_(1,29)_ = 8.32, *p* = 0.007, η_p_^2^ = 0.22), indicating that tone changes elicited larger MMN responses in tonal language speakers. More importantly, there was a significant interaction between TMS and group (*F*_(1,29)_ = 8.57, *p* = 0.007, η_p_^2^ = 0.23; see Fig. 3*B*; the main effect of TMS was not significant: *F*_(1,29)_ = 4.04, *p* = 0.061, η_p_^2^ = 0.12). *Post hoc* paired *t* tests indicated that the disruption of the right speech motor cortex suppressed MMN responses to the tone change in the non-tonal (*t*_(14)_ = 3.41, *p* = 0.004, *d* = 0.88) but not in the tonal language group (*t*_(14)_ = 0.65, *p* = 0.526, *d* = 0.16).

In sum, our results showed that in both language groups, TMS-induced disruption of the right speech motor cortex had no significant effect on MMN responses elicited by a phoneme change. In contrast, the effect of the right-hemisphere disruption on MMN responses elicited by a tone change differed according to language experience: it significantly suppressed MMN responses in the non-tonal language group but had no effect on MMN responses in the tonal language group.

### Effects of TMS-induced disruption in the left speech motor cortex

We stimulated the left speech motor cortex in another group of 15 non-tonal language speakers and a further 16 tonal language (Mandarin) speakers and tested the effect of this disruption on discrimination of tone and phoneme contrasts measured by the MMN. Data were analyzed as described for the right motor cortex.

**Figure 4.**
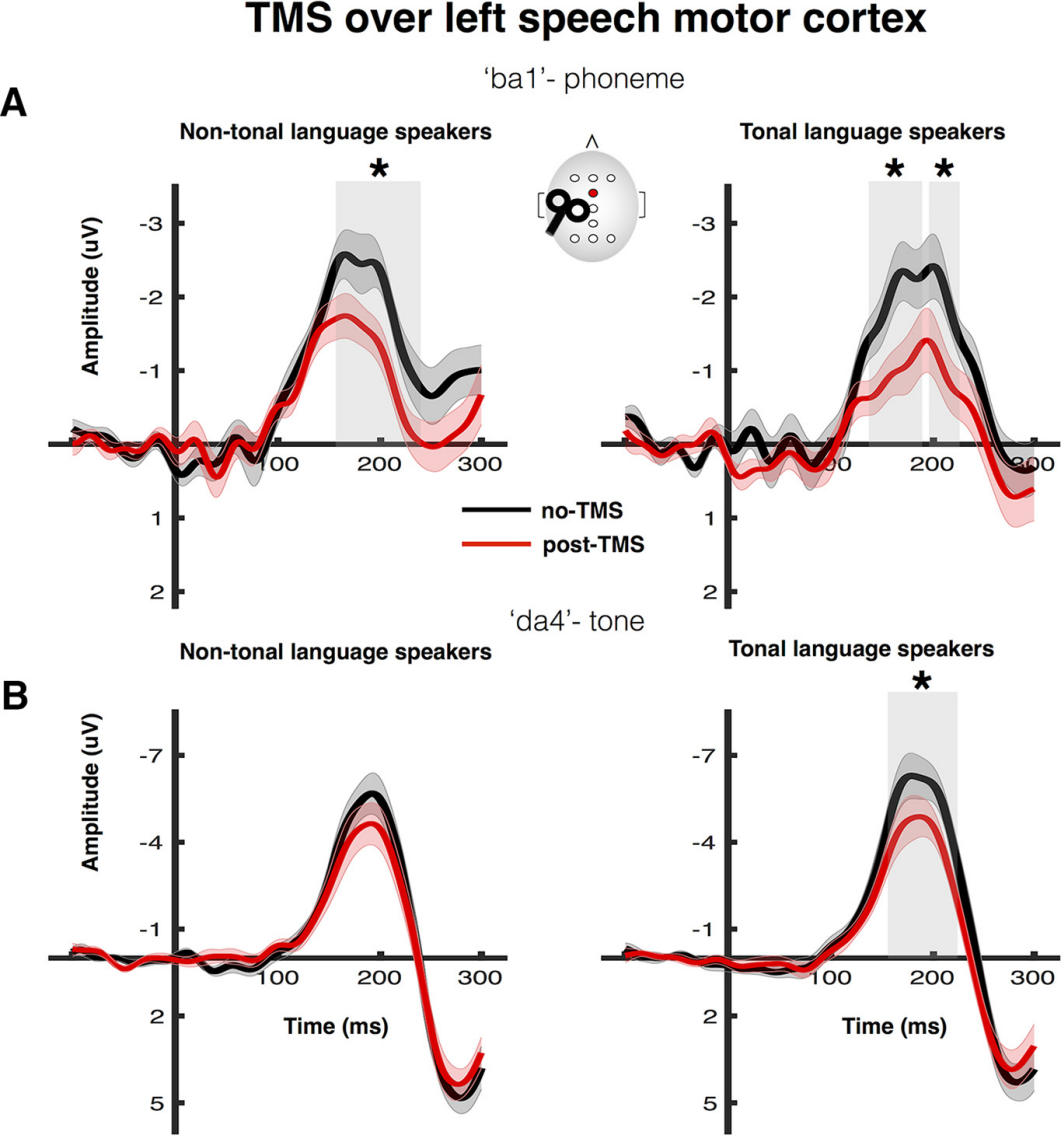
Effect of rTMS over left speech motor cortex on MMN responses elicited by the phoneme (***A***) and tone (***B***) changes in tonal and non-tonal language speakers. See legend to Figure 2 for details.

TMS-induced disruption of left speech motor cortex significantly suppressed the amplitude of the MMN responses elicited by the phoneme change in both the non-tonal and the tonal language groups. These differences were significant at a latency of 162–224 ms after stimulus onset in the non-tonal language group and at latencies of 134–190 and 196–224 ms after stimulus onset in the tonal language group (Fig. 4*A*, 'ba1'). In contrast, MMN responses elicited by the tone change were significantly suppressed by TMS disruption of left speech motor cortex in the tonal language group at a latency of 154–224 ms after stimulus onset (Fig. 4*B*, 'da4'); in the non-tonal group, the slight suppression of MMN responses to the tone change was not significant at any latency (Fig. 4*B*, 'da4').

Analysis of the peak amplitudes (one-sample *t* test against zero) confirmed that significant MMN responses were elicited in both the no-TMS and post-TMS sessions by the phoneme and the tone changes in both groups (Table 1). Comparison of MMN responses calculated using the traditional method with identity MMNs calculated using the same-stimulus method confirmed (as above for the groups who received right hemisphere stimulation) that these changes were because of discrimination of speech sounds (Tables 1, 2). Furthermore, during the no-TMS session, we confirmed that the latencies and amplitudes of the MMN responses elicited by either tone or phoneme changes did not differ between language groups (two-sample *t* tests: all *p* > 0.05).

**Figure 5.**
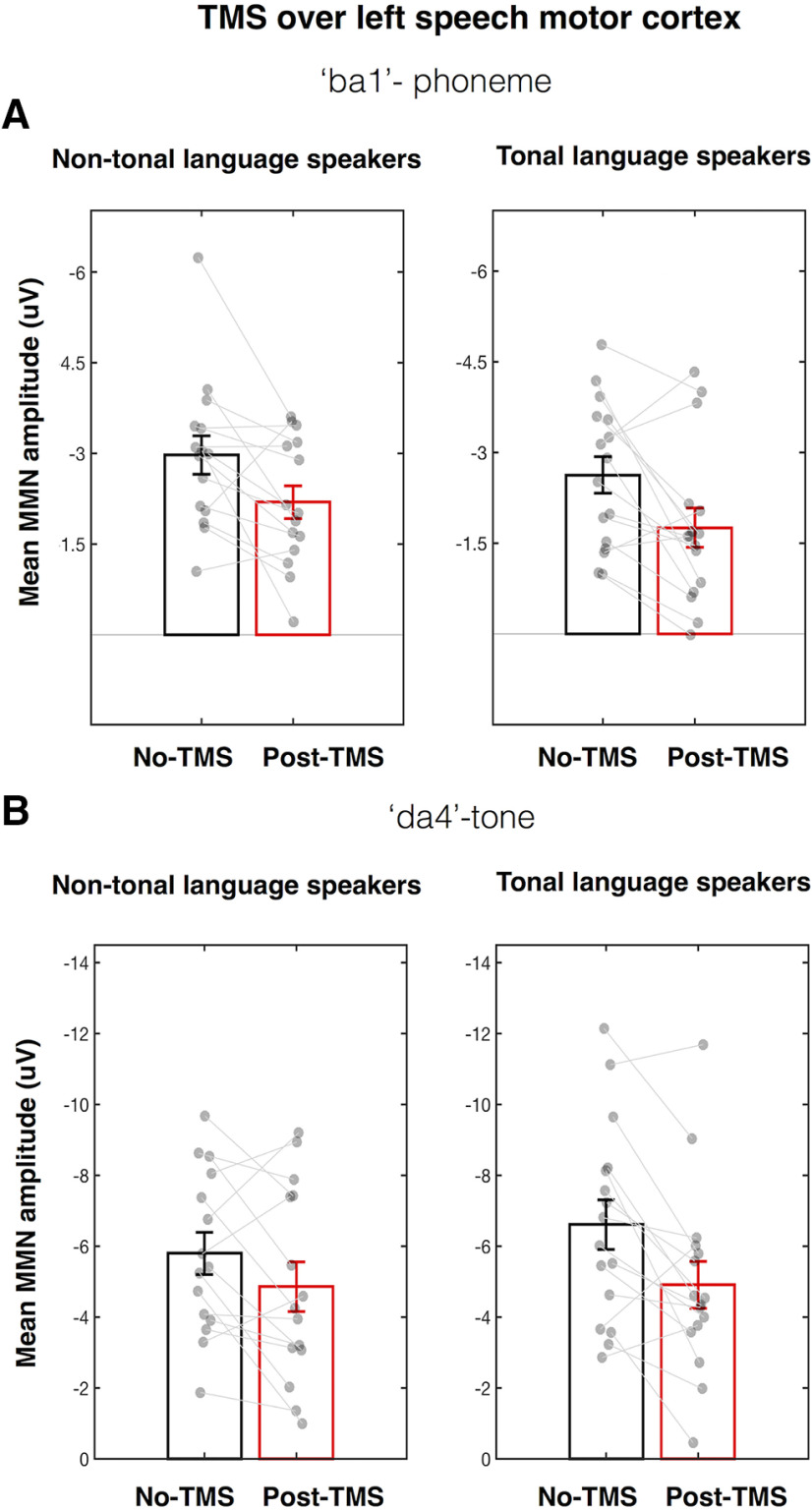
Effect of rTMS over the left speech motor cortex on amplitudes of MMN responses elicited by the phoneme (***A***) and tone (***B***) changes in tonal and non-tonal language speakers. See legend to Figure 3 for details.

To test whether the effects of TMS-induced disruption of left speech motor cortex on the MMN responses were sensitive to the language experience of the participants, we compared the groups using two-way ANOVAS for the phoneme and tone contrasts separately. For the MMN responses elicited by the phoneme change, the TMS-induced disruption in the left speech motor cortex significantly suppressed MMN responses in both non-tonal and tonal language groups (main effect of TMS: *F*_(1,29)_ = 15.56, *p* < 0.001, η_p_^2^ = 0.35; see Fig. 5*A*; the main effect of group was not significant: *F*_(1,29)_ = 1.09, *p* < 0.306, η_p_^2^ = 0.04). Furthermore, the magnitude of the suppression did not differ between the groups (interaction: *F*_(1,29)_ = 0.05, *p* = 0.823, η_p_^2^ = 0.002). For the MMN responses elicited by the tone change, the TMS-induced disruption in the left speech motor cortex significantly suppressed peak amplitudes in both groups (main effect of TMS: *F*_(1,29)_ = 13.80, *p* = 0.001, η_p_^2^ = 0.32; see Fig. 5*B*; the main effect of group was not significant: *F*_(1,29)_ = 1.14, *p* < 0.294, η_p_^2^ = 0.04). The suppression in the tonal language group was numerically larger (mean MMN peak amplitude change ± SE = 1.70 ± 0.50 µV, *d* = 0.85) compared with the size of the suppression in the non-tonal language group (mean MMN peak amplitude change ± SE = 0.94 ± 0.50 µV, *d* = 0.48; see Fig. 5*B*) but this group difference was not statistically significant (TMS × group interaction: *F*_(1,29)_ = 1.14, *p* = 0.294, η_p_^2^ = 0.04).

In sum, our results showed that in both language groups TMS-induced disruption of the left speech motor cortex suppressed MMN responses elicited by a phoneme change. In contrast, the effect of left-hemisphere disruption on MMN responses elicited by a tone change differed according to language experience: the reduction in the non-tonal language group was not significant at any latency (Fig. 4*B*) whereas it was significantly reduced at 154- to 224-ms latency in the tonal language group. However, the analysis of the peak amplitude MMN responses to tone discrimination during disruption of the left speech motor cortex found that the non-significant small reduction in the non-tonal language group was statistically indistinguishable from the significant and large effect seen in the tonal language group (i.e., the interaction between group and TMS was not significant, *p* > 0.05). Examination of data shown in Figure 5*B* indicates that some individuals in the non-tonal language group showed large reductions in the MMN amplitude elicited by the tone change ('da4'), but this was not a reliable effect across the group.

## Discussion

We used EEG and TMS to investigate the asymmetry of auditory-motor processing of phoneme and tone changes in speakers of tonal and non-tonal languages. Disruption of the left, but not the right, speech motor cortex impaired automatic auditory discrimination of phonemes in both tonal and non-tonal language groups. Disruption of the right speech motor cortex modulated auditory discrimination of tones in the non-tonal language group but had no effect in the tonal language group. Tone discrimination in the tonal language group was affected by disruption of the left speech motor cortex. The effect of disruption of the left motor cortex on tone discrimination in the non-tonal language group was inconclusive: in one analysis, the MMN responses to tone changes were not significantly reduced by the TMS disruption at any latency, whereas the analysis of changes in the peak amplitude found that the small suppression in the non-tonal language group was not statistically different to the larger suppression seen in the tonal language group. This is the first study to provide direct evidence that the lateralization of motor contributions to auditory speech processing is determined by the functional role of the acoustic cues in the listener's native language.

Our finding that disruption of the left motor cortex impaired auditory processing of both phoneme and tone changes in tonal language speakers is consistent with findings from previous studies. For example, early lesion studies with patients reported impaired lexical tone production and perception in tonal language speakers following damage to the left hemisphere (Gandour et al., 1992; Yiu and Fok, 1995; Eng et al., 1996). Later, functional neuroimaging data also revealed that in tonal language speakers processing of lexical tone evoked activity in several left-hemisphere regions, including the left posterior temporal cortex (Gandour et al., 2000, 2003; Klein et al., 2001; Pierce et al., 2014).

In the current study, disruption of the right speech motor cortex impaired tone discrimination in non-tonal language speakers. As for the findings in tonal language speakers, these results are consistent with a known pattern of right hemisphere lateralization in non-tonal language speakers for tone and prosody perception based on findings from the lesion deficit (Zatorre and Samson, 1991) and neuroimaging literature (Boemio et al., 2005; Schönwiesner et al., 2005; Albouy et al., 2020).

The motor contribution to auditory tone processing was strongly left-lateralized in tonal language speakers, while it was weakly right-lateralized in non-tonal language speakers. These findings are consistent with previous work indicating that processing of spectral modulations was weakly right lateralized compared with a strong left lateralization for processing of temporal modulations (Flinker et al., 2019). We found that the MMN responses elicited by tone changes in tonal language speakers were unchanged following right hemisphere motor disruption. For the non-tonal language speakers, right hemisphere motor disruption reduced MMN responses to tone changes by a significant amount but there was also a small reduction in these responses following left-hemisphere motor disruption. This effect of left-hemisphere disruption was not observed in a previous study of non-tonal language speakers, in which behavioral discrimination of prosody in spoken questions and statements was impaired by disruption of right but not left premotor cortex (Sammler et al., 2015). It is possible that use of the MMN offers greater sensitivity compared with behavioral measures of task performance to reveal the subtle effect of left-hemisphere disruption in the non-tonal language speakers, which was considerably smaller than the effect of right-hemisphere disruption. One advantage of using the MMN is that it provides an automatic measure of auditory discrimination and, unlike task performance, does not require the listener to attend and respond to the relevant features of the sounds. Such task demands could interact with TMS-induced effects on auditory processing. It is possible that the auditory-motor processing of tone in speech is bilaterally organized with a weak right-hemisphere bias in non-tonal language speakers. However, the focus of attention on tones could enhance auditory-motor processing in the right hemisphere leading to increased lateralization.

The current study confirms the contribution of speech motor cortex to auditory processing of phoneme and tone changes in both tonal and non-tonal language speakers. The findings therefore provide further evidence for the causal role of the speech motor cortex in speech processing (Davis and Johnsrude, 2007; Möttönen et al., 2013). Note that our results provide support for the critical role of speech motor cortex in speech perception but not speech or word recognition. It is important to be aware that although the syllables used in the current study ('da1,' 'da4,' 'ba1') have “lexical significance” in Mandarin, it is impossible to determine their meaning by listening to them in isolation. For example, the syllable 'da1' could mean “build,” the sound of a horse's hooves, “floppy,” and tens of other meanings dependent on the context. Thus, for both non-tonal and tonal language speakers, passive listening to the oddball sequences involved automatic discrimination of the syllables (i.e., sublexical processing) rather than access to meaning. The complementary contribution of speech motor cortex to speech perception might be observed only when contextual information is unavailable (Wilson, 2009); this could partially reconcile findings from studies in patients showing relatively intact speech comprehension after damage to frontal-motor brain areas (Hickok et al., 2008; Hickok, 2010). In real life conditions, listeners, including patients with Broca's aphasia, can make use of contextual information to “comprehend” words even when their speech perception is partially impaired.

The auditory-motor processing of speech sounds observed in the current study was not feature specific: the disruption of speech motor cortex involved targeting the lip representation separately in each hemisphere. The disruption affected both phoneme and tone discrimination, even though tone production is controlled by the larynx. This is consistent with our previous work showing that disruption of the left lip but not the left hand representation suppressed MMN responses evoked by infrequent lip-related 'ba' and tongue-related 'ga' sounds presented among frequent tongue-related 'da' sounds (Möttönen et al., 2013); the disruption had no effect on discrimination responses for non-speech sounds. However, previous behavioral studies suggested that the contribution of the motor cortex to speech perception was somatotopically organized (D'Ausilio et al., 2009; Möttönen and Watkins, 2009). The non-specific effect observed here could be because of the close proximity of the lip and larynx representations, which makes it impossible to disrupt the lip without affecting the adjacent larynx representation (Grabski et al., 2012; Eichert et al., 2020). An alternative explanation is that the feature-specific motor contributions to speech perception depend on attention. We showed previously that when speech sounds are ignored, the motor contributions to auditory speech processing are late (>170 ms) and not feature specific, whereas when speech sounds are attended, there is an additional early (<100 ms) motor effect on auditory speech processing, which is feature specific (Möttönen et al., 2014b).

Our findings lead us to suggest that the asymmetry in the auditory system during speech perception is modulated by the motor system. Recently, a modified version of the asymmetric-sampling-in-time hypothesis was proposed, in which auditory processing is thought to be modulated by two different types of mechanism (Rimmele et al., 2018; Assaneo et al., 2019): (1) intrinsic auditory mechanisms, which support auditory processing in non-speech listening conditions in line with acoustic hypotheses; and (2) top-down mechanisms, which might affect the lateralization of auditory processing, and the relative weight of which can increase depending on individual brain differences or specific speech-listening conditions or both. During continuous speech perception, the speech-coupled oscillations in auditory cortex are significantly modulated by top-down signals from the frontal and motor cortex (Park et al., 2015). This might partially account for left-lateralized auditory speech processing that is determined by linguistic experience and the functional role of acoustic cues. A recent study in non-tonal language speakers investigated the hemispheric asymmetry of auditory-motor interactions during speech feedback control and found that the asymmetry was affected by spectro-temporal cues (Floegel et al., 2020). Our results predict that language experience and the functional role of the spectro-temporal cues may also affect the involvement of left and right auditory-motor interactions in speech feedback control during speech production.

In sum, our study, for the first time, presents causal evidence that the contribution of the right and left speech motor cortex to auditory speech discrimination ability is determined by the functional role of acoustic cues in the listener's native language. This motor system asymmetry could modulate the asymmetry in the auditory system. Our study emphasizes the importance of understanding the interactions between bottom-up auditory mechanisms and top-down influences when investigating hemispheric asymmetry of speech processing. We also call for more comparative studies between speakers of different languages, since ignoring the diversity of spoken languages can lead to an incomplete understanding of the neurobiology of speech and language.
